# AlignMiner: a Web-based tool for detection of divergent regions in multiple sequence alignments of conserved sequences

**DOI:** 10.1186/1748-7188-5-24

**Published:** 2010-06-02

**Authors:** Darío Guerrero, Rocío Bautista, David P Villalobos, Francisco R Cantón, M Gonzalo Claros

**Affiliations:** 1 Plataforma Andaluza de Bioinformática (Universidad de Málaga), Severo Ochoa, 34, 29590 Málaga, Spain; 2 Departamento de Biología Molecular y Bioquímica (Universidad de Málaga), Campus de Teatinos, 29071 Málaga, Spain

## Abstract

**Background:**

Multiple sequence alignments are used to study gene or protein function, phylogenetic relations, genome evolution hypotheses and even gene polymorphisms. Virtually without exception, all available tools focus on conserved segments or residues. Small divergent regions, however, are biologically important for specific quantitative polymerase chain reaction, genotyping, molecular markers and preparation of specific antibodies, and yet have received little attention. As a consequence, they must be selected empirically by the researcher. AlignMiner has been developed to fill this gap in bioinformatic analyses.

**Results:**

AlignMiner is a Web-based application for detection of conserved and divergent regions in alignments of conserved sequences, focusing particularly on divergence. It accepts alignments (protein or nucleic acid) obtained using any of a variety of algorithms, which does not appear to have a significant impact on the final results. AlignMiner uses different scoring methods for assessing conserved/divergent regions, Entropy being the method that provides the highest number of regions with the greatest length, and Weighted being the most restrictive. Conserved/divergent regions can be generated either with respect to the consensus sequence or to one master sequence. The resulting data are presented in a graphical interface developed in AJAX, which provides remarkable user interaction capabilities. Users do not need to wait until execution is complete and can.even inspect their results on a different computer. Data can be downloaded onto a user disk, in standard formats. *In silico *and experimental proof-of-concept cases have shown that AlignMiner can be successfully used to designing specific polymerase chain reaction primers as well as potential epitopes for antibodies. Primer design is assisted by a module that deploys several oligonucleotide parameters for designing primers "on the fly".

**Conclusions:**

AlignMiner can be used to reliably detect divergent regions via several scoring methods that provide different levels of selectivity. Its predictions have been verified by experimental means. Hence, it is expected that its usage will save researchers' time and ensure an objective selection of the best-possible divergent region when closely related sequences are analysed. AlignMiner is freely available at http://www.scbi.uma.es/alignminer.

## Background

Since the early days of bioinformatics, the elucidation of similarities between sequences has been an attainable goal to bioinformaticians and other scientists. In fact, multiple sequence alignments (MSAs) stand at a crossroad between computation and biology and, as a result, long-standing programs for DNA or protein MSAs are nowadays widely used, offering high quality MSAs. In recent years, by means of similarities between sequences and due to the rapid accumulation of gene and genome sequences, it has been possible to predict the function and role of a number of genes, discern protein structure and function [1], perform new phylogenetic tree reconstruction, conduct genome evolution studies [2], and design primers. Several scores for quantification of residue conservation and even detection of non-strictly-conserved residues have been developed that depend on the composition of the surrounding residue sequence [3], and new sequence aligners are able to integrate highly heterogeneous information and a very large number of sequences. Without exception, the sequence similarity of MSAs is optimised [4]. Some databases such as Ensembl and PhIGs can provide information on conserved regions across different species.

In contrast, meanwhile, detection of divergent regions in alignments has not received the necessary attention, with the inevitable consequence of a lack of appropriate tools to address this subject. Divergent regions are in fact as biologically interesting as similar regions, since they are useful in the following aspects: (i) high-throughput expression profiling using quantitative PCR (qPCR), which is considered to distinguish between closely-related genes [5]; (ii) confirmation of expression results obtained by microarray technology, as well as quantification of low-abundance transcripts; (iii) taxonomy and varietal differentiation is based on small differences between organisms: it enables appropriate categorisation. Since the genetic material of individuals from the same species is very similar, it is necessary to detect specific differences to distinguish between them [6]; (iv) SNP (single nucleotide polymorphism) and diseases: most differences between healthy and unhealthy organisms are based on single-nucleotide differences [7]; (v) identification of pathological and autopsy specimens in forensic medicine is based on minimal sequence differences among samples [8]; (vi) primer design for PCR-based molecular markers relies on differences among DNA sequences [9]; (vii) one way of preparing highly-specific monoclonal antibodies is by immunisation with highly-divergent peptides, instead of with the whole protein [10].

Analysis of gene and genomic variation has been revolutionised by the advent of next-generation sequencing technology, revealing a considerable degree of genomic polymorphism. This has led to studies focusing on SNP discovery and genotyping [7,11-18], as well as the design of properly conserved primer candidates from MSAs [19,20], for comparative studies of genes and genomes [21]. Most of these tools are operating system-dependent and only a few are Web-based, in which case they have a relatively static interface. However, there is neither adequate software for, nor study on, MSAs for detection of polymorphic regions and discrepancies (beyond single nucleotide dissimilarities) that would provide a numerical score related to divergence significance. In short, researchers find themselves empirically detecting which sequence fragment, among a series of paralogs and/or orthologs, can be used to design specific primers for PCR, or which specific probes or specific linear epitopes can be synthesised in order to obtain antibodies. Together, these factors have been the main motivation for development of AlignMiner: this software was intended to cover the gap in bioinformatic function by evaluating divergence, rather than similarity, in alignments that involve closely-related sequences. For any type of DNA/protein alignment, through its Web interface AlignMiner is able to identify putative SNPs, divergent regions, and conserved segments. The results can be inspected graphically via an innovative, interactive graphical interface developed in AJAX, or saved in any of several formats.

## Implementation

### Architecture

AlignMiner is a free Web-based application that has been developed in three layers, each making use of object-oriented methodologies. The first layer contains the algorithm core. It is written entirely in Perl and uses Bioperl [22] libraries for MSA loading and manipulation. Hence, it can run in any operating system provided that Perl 5.8, BioPerl 1.5.2, and the Perl modules Log::Log4perl, JSON and Math:FFT are installed. BioPerl has been chosen because it provides a rich set of functions and an abstraction layer that handles nearly all MSA formats currently available. The second layer links the algorithm with the interface using the necessary CGIs written in Perl. The third (top) is a front-end layer based on AJAX [23] techniques to offer an interactive, quick and friendly interface. Intermediate data and final results are saved using JSON [24], a data format that competes with XML for highly human-readable syntax, and for efficiency in the storage and parsing phases. Firefox or Safari Web browsers are recommended, since Internet Explorer does not support some of the advanced features of AJAX. AlignMiner has been tested for correct operation in a few flavours of Linux and various Mac OS X machines, to verify full compatibility.

Owing to its layered architecture, AlignMiner can function in four execution modes: (i) as a command line for advanced users to retain all **Unix** capabilities of integration, within any automation process or pipeline; (ii) as a REST Web service, also for advanced users, which enables its integration in workflows; (iii) as a single workstation where jobs are executed on the same computer that has the Web interface -- this setting is not recommended since it is prone to saturation when multiple jobs are sent simultaneously; (iv) as an advanced Web application (this is the preferred mode), where jobs are transferred to a queue system which schedules the execution depending on the resource availability -- this minimises the risk of saturation while maintaining interactivity. Data management remains hidden to users.

### Algorithm

**Figure 1:**
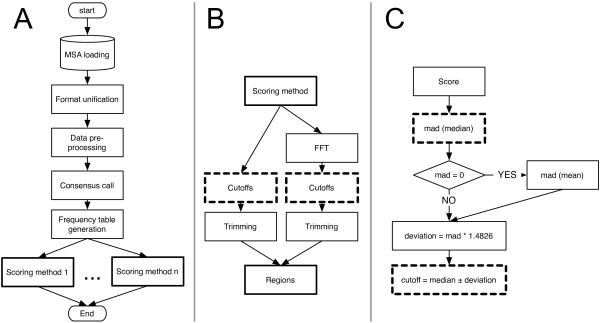
**The AlignMiner algorithm**. (A) Flow diagram of the main components of the algorithm, as explained in the text; the bold boxes are detalied in B. (B) The details of how a divergent region is obtained using a given scoring method. The "score calculation" renders a single numeric value for each MSA column. "FFT" is a fast Fourier transform for smoothing the curve of raw scores. The original (left branch) and Fourier-transformed (right branch) curves are trimmed with their respective "cutoffs" in order to obtain putative SNPs and conserved/divergent regions, respectively. The bold dashed boxes are detailed in C. (C) Details of the determination of the final cutoffs used for trimming scores and providing the validated conserved/divergent regions.

The AlignMiner algorithm is outlined in Figure 1A. It can be divided into the following main steps:

1. **Sequence or MSA loading: **Since AlignMiner is not intended to build the best possible MSA, users are expected to load already-built MSAs obtained using external programs such as M-Coffee [25,26] or MultAlin [27] (for a review of MSA tools, see [4]). However, AlignMiner is also able to align a set of sequences in FASTA, MSF, CLIJSTALW and other formats using the fast, accurate and memory-efficient Kalign2 [28]. The alignment file is loaded into the Bioperl SeqIO abstraction object, which enables AlignMiner to read nearly all MSA formats. The format is not inferred from the file extension but by searching the file contents for format-specific patterns. Users are alerted if there are faulty, corrupted or unknown file formats.

2. **Format unification: **For efficient data management, all MSA formats are encapsulated into a common JSON representation and saved to disk to make them accessible to other AlignMiner modules.

3. **Data pre-processing: **The alignment is examined to extract basic characteristics that are used in internal decisions, such as the number of sequences, MSA length, type of aligned sequences (DNA/protein), and MSA format, and an identifier is assigned to each sequence. These characteristics are also displayed in the 'Job List' tab in order to provide some information regarding the MSA content. Finally, AlignMiner automatically analyses the MSA to determine the region where the algorithm is going to be applicable: there is usually a high proportion of gaps at each MSA end that would lead to misleading results for frequencies (see below), due to the small number of sequences and the low alignment reliability at these positions [1]. The MSA ends are then sliced until at least two contiguous positions do not include any gap. Slicing limits can also be set manually if desired.

4. **Consensus call: **A consensus sequence is assessed from the whole MSA using BioPerl capabilities to serve as the weighting reference for calculations. When a user defines one sequence within the MSA as the **master** sequence, scoring calculations (see below) will now be referred to it instead of to the consensus.

5. **Frequency table: **Since the scores implemented in AlignMiner require knowledge of the number of nucleotides or amino acids present at each position of the MSA, these frequencies are stored in temporary tables as a simple caching mechanism to speed up the algorithm performance, in order to spend nearly the same time with a few aligned sequences as with a large number of aligned sequences (see below).

6. **Scoring: **Several scoring methods (see next section for details) are included in AlignMiner in order to enhance different aspects of each MSA. This is the slowest portion of the algorithm since each scoring method has to read and process the complete MSA (further optimisation, including parallelisation, will be addressed to this step in the near future). Each scoring method provides a single value for each alignment column that enables the evaluation of conservation (positive value) or divergence (negative value) at every column of the MSA (Figure 1B). Concerning gaps, there is neither consensus interpretation nor an adequate model for handling gaps in alignments. Therefore, in this work, the presence of a gap in a column is considered as the lowest conservative substitution. By default, it is expected that sequence divergence is spread over the sequence (as was previously with the case with protein MSAs), such that scores produce clear maximum and minimum peaks reflecting conserved and divergent positions, respectively. In order to extract the significant peaks, a robust and consistent measure is calculated based on the median value of the score and two cutoffs (Figure 1C). Cutoffs rely on 1.4826 times the *median absolute deviation (MAD *= *median[abs(X – median*[*X*])]) such that they define a margin equivalent to one standard deviation from the median. When sequences in the MSA are closely related (note that DNA sequences are to be closely related), the median is 0, and the MAD is also 0 or very close to 0. In such a case, a reliable cutoff was established using a MAD-like measure based on the mean (instead of the median) to avoid the overpopulation of zero-valued positions, such as *MAD_mean *= *mean*[*abs(X - mean*[*X*])]. This cutoff will only reveal divergent regions of the MSA.

7. **Regions: **Nucleotides whose score is below the low cutoff boundary are reported as a putative SNP provided that each variation appears in at least two sequences (as a consequence, alignments of less than four sequences would lack the capacity for SNP prediction). It should be taken into account that neither synonymy nor the potential effects on protein structure are checked for these putative SNPs, since AlignMiner is not designed to predict the significance of SNPs. Obviously, such a calculation is not performed with protein MSAs. Raw scores are smoothed by a fast Fourier transform ("FFT" in Figure 1B) such that contiguous sharp peaks become wide ranges in order to assess changes in regions, rather than nucleotides. The algorithm reports those positions of the raw and FFT-transformed values that have a score higher (conserved) or lower (divergent) than the corresponding cutoffs for conserved/divergent regions. In the case of DNA alignments, divergent regions must additionally include at least two putative SNPs. The arithmetic mean of the score of every nucleotide/amino acid encompassed by that region gives the characteristic score for the region.

#### Scoring methods

All scoring methods described below are included in the common base algorithm depicted in Figure 1, since they are all based on the information contained in each column of MSAs. The only differences between the scoring methods are in the weight table and formula for each. All scores are calculated specifically for each type of sequence (DNA/protein) and for the particular MSA being processed, so it is up to users to decide which one best applies in their situation. Common parameters for all scoring methods are:

• *γ*(*i, b*) → Count of nucleotide instances *b *at position *i *of the MSA.

• *C*(*i*) → Nucleotide at position *i *in the consensus or master sequence.

• *M*(*b*1, *b*2) → Weighting for nucleotide *b*2 when its corresponding *C*(*i*) is *b*1.

• *D*(*i*) → Number of different nucleotides found at position *i *of the MSA.

• *B *→ Set of nucleotides found in the MSA.

• *nseq *→ Number of sequences in the MSA.

It should be taken into account that each of the following scoring methods will provide a different score range. However, all of them are intended to produce positive values for conserved regions and negative values for divergent regions, and are not zero-centred in any case.

##### Weighted

The Weighted score is applicable to any sequence type. For each position *i*of the alignment, it is calculated as:(1)

A weight matrix [29,30] is used for promoting identities over similarities, and penalising (giving a negative value) to the differences depending on the degree of divergence. Accordingly, the result is not zero-centred unless aligned sequences were quite different. It is not expected that changing the weight matrix would produce significant differences. Matrices for DNA alignments are taken from WU-Blast (Warren R. Gish, unpublished): "Identity" is given for sequences with only the four usual nucleotides (ACTG), and "Simple" for sequences including undefined nucleotides (RYMWSK). Protein alignments are weighted using "Blosum62" [31,32].

##### DNAW

DNAW applies only to DNA sequences containing A, C, T and G, since it is a simplification of the Weighted score when weights are 1 for identity and 0 for difference. Hence, for each position i of the alignment,(2)

As a result, and like Weighted, a lower value is obtained when the difference found between sequences is higher. Again, it is not zero-centred.

##### Entropy

A parameter frequently used for quantifying the composition of an individual column i is its entropy *H(i)*, since it is an ideal representation of disorder at every MSA position and can be very usefully employed to assess differences in a MSA. *H*(*i*) is defined as follows (using frequencies instead of probabilities):(3)

However, for consistency with the rest of the scoring results (where divergent regions are represented with lower values than conserved ones), Entropy scoring is sign-switched, such that *Entropy *= --*H*(*i*).

##### Variability

Variability represents another way to evaluate changes in an alignment position without taking into account whether variations are conservative or not. The rationale is that any position change is valid for marking a difference between sequences. Negative values indicate greater variability. It is defined by the equation:(4)

#### Primer design module

One of the most useful applications derived from retrieval of divergent regions is the design of PCR primers "on the fly". A window containing the divergent region plus five nucleotides on each side defines a primer by default. Parameters for the displayed nucleotide window are calculated as in [33], that is: length, GC content, melting temperature, absence of repeats and absence of secondary structures. An optimal primer sequence should contain: (i) two to three G's or C's for 3'-end stability; (ii) a GC content of between 40% and 60%; (iii) a melting temperature above 52°C; and (iv) the absence of secondary structure formation, that is, the maximum free energy must be above -4 kcal/mol for dimer formation or -3 kcal/mol for hairpin formation. Every parameter is printed over a colour that suggests the value compliance: green indicates that the primer is in agreement with the above requirements, and orange, red or blue that the sequence should be optimised. Users can move the window size in order to obtain optimal parameters so that the optimal primers are expected to have "green" properties (Additional file 1 Figure S1). The primers so designed can be tested *in silica *by means of the "PCR amplification" Web tool [34] at BioPHP [35] against every sequence of the alignment. It should be noted that primers designed with AlignMiner are intended to identify a specific sequence; therefore, degenerate primer design is disabled.

#### Usage

The AlignMiner Web interface was designed for maximum simplicity and convenience of use. Users must log on with their e-mail to obtain a confidential space within the public environment (no registration is needed). Their data are stored there for at least four weeks, although old jobs may be deleted by the administrator for space limitation reasons; in fact, users are recommended to locally save their analysis. A new job starts when a file containing one MSA (most popular formats are accepted such as Clustal, NEXUS, MSF, PHYLIP, FASTA...), or a set of sequences to be aligned with Kalign2, is uploaded and a name is optionally given. A small amount of basic information (sequence count, length, file type, etc) about every job is shown to the user in order to verify that it has been correctly pre-processed. Users can then decide to mark a specific sequence as master. In such a case, the algorithm is directed to look for the most divergent/conserved regions with respect to the master instead of the consensus sequence. This option enables identification of overall divergences (by default) or regions that serve to clearly differentiate the master sequence from the other sequences. Finally, users can either decide themselves which portion of the alignment will be analysed, or allow AlignMiner to decide.

At this moment, the job is already shown in the Job List with a "waiting" status. Once the "Run" button is pushed, the batch system takes control, and the status (pending, queued, running or completed) is displayed in real time. Afterwards, users can decide to (1) wait until the most recent job is finished, (2) browse previously-completed jobs, (3) launch new jobs, or (4) close the Web browser and return later (even on a different computer) to perform any of the first three operations. Job deletion is always enabled.

By clicking on each job, users can select a scoring method for analysis of their MSA. Changing the scoring method is always comparatively fast, since calculations have already been performed. Results are shown in a dynamic display that enables clicking, scrolling, dragging, zooming, and even "snapshooting" a portion of the graphical plot. The plot can be saved on the user's computer in PNG format; a record of snapshots is additionally maintained on the screen. Results are also represented in a tabular form linked to the graphical plot: each table row is linked to its corresponding region in the plot, and vice-versa. Tables can be ordered by position or score values, and exported to GFF (general features format) for external processing.

AlignMiner can also be used as a Web service. The REST protocol has been used due to is wide interoperability and because it only needs an HTTP stack (either on the client or the server) that almost every platform and device has today. The Web service of AlignMiner can be invoked to send, list, delete, or download jobs. Job results can be downloaded as a whole, or file by file. URL, http verb and optional fields are indicated in Additional file 2 Table S1. The api_login_key field is compulsory for any REST invocation of AlignMiner since it serves to allocate the corresponding disk space. An example of submitting a new job using the curl client is:

curl -X POST

   -F http://api_login_key=your@email.net

   -F alignment_file_field=@/tmp/tests/sequences.fna

   -F job_name_field=MyAMtest

   -F master_field=NONE

   -F align_start_field = 0

   -F align_end_field = 0

   http://www.scbi.uma.es/ingebiol/commands/am/jobs/0/stage/1.json

Obtaining a job status by means of a browser is performed by:

http://www.scbi.uma.es/ingebiol/commands/am/jobs/20100412.json?api_login_key=your@email.com

### Polymerase chain reaction

Each PCR was performed on a T1 Thermocycler (Biometra). The PCR reaction mixture for a 100 *μ*l volume contained 75.5 *μ*l of distilled water, 10 *μ*l 10 × PCR buffer, 2 *μ*l dNTP mix (12.5 mM each), 2 *μ*l of each primer (20 *μ*M), 0.5 *μ*l *Taq *polymerase (5 U/*μ*l), and 5 *μ*l of template DNA. The PCR commenced with 5 min of denaturation at 94°C and continued through 35 cycles consisting of the following steps: 94°C for 1 min, 4°C over the lowest melting temperature (Tm) of the corresponding primer pair for 1 min, and 72°C for 2 min. Cycles were followed by a final extension step at 72°C for 8 min. When the template was cDNA or plasmid DNA, the 5 *μ*l of template contained 20 ng of DNA, whereas it contained 1 *μ*g when template was genomic DNA. The amplification products were analysed using 1.5% (w/v) agarose gel electrophoresis.

## Results and Discussion

The vast amount of data involved in MSAs makes it impossible to manually identify the significantly divergent regions. In order to assess the speed, success rate and experimental usefulness of AlignMiner with different real and hypothetical MSAs, two algorithms for MSA were used: one is M-Coffee [25] which generates high-quality MSAs by combining several alternative alignment methods into one single MSA, and the other is MultAlin [27] which is based on a hierarchical clustering algorithm using progressive pairwise alignments.

### AlignMiner Performance

**Figure 2:**
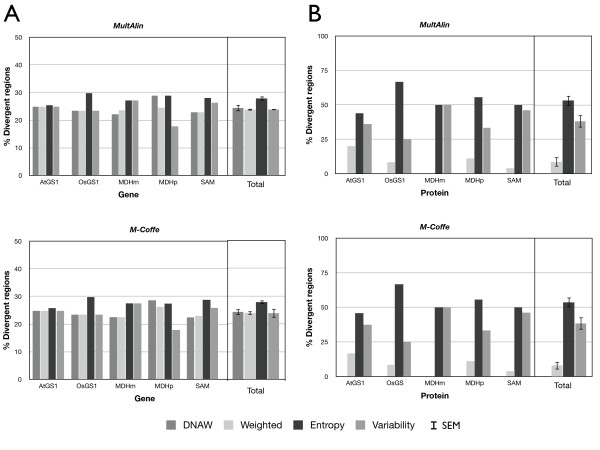
**Execution time versus number of nucleotides in the MSA, excluding delays due to the queue system**. The upper panel represents the time taken when MSA length increases for a given number of sequences. The lower panel (solid line) represents the time taken when MSA length is kept constant while the number of sequences is increased. The number of nucleotides in each case is a simple multiplication of MSA length by the number of sequences.

The speed and performance of AlignMiner were analysed by increasing the two-dimensional size of the MSA. A first assay was designed to test AlignMiner performance when increasing the number of aligned sequences for a fixed length. The second test was designed to assess AlignMiner behaviour when a fixed number of sequences (four in this case) contained longer and longer alignments. Figure 2 clearly shows that execution time increased with the number of nucleotides included in the MSA. However, it was not significantly affected by the number of aligned sequences (solid line), but by the increase in alignment length (dashed line). Accordingly, the execution time would be long only when relatively long genomic sequences were analysed. This behaviour was expected, since AlignMiner is optimised to work with an extremely large number of sequences through the use of frequency tables (see the Algorithm section).

These caching techniques allowed the algorithm to use the same amount of memory and spend a fixed time in score calculation, independently of the number of sequences loaded. The subtle increment in time related to the increment in sequences arises from population of the frequency table, which was done sequentially for every aligned sequence.

Computationally, these assays provided further information for AlignMiner, since they were executed on a multiprocessor computer where the queue system was to be given some information regarding the estimated execution time for each job. Obviously, it is impossible to provide an exact value in every case, but the execution times shown in Figure 2 served to provide an estimated execution-time curve for the queue system.

### Scoring method characterisation

Since the rationale of each scoring method is different, they must be characterised in order to know when each particular method is more appropriate. Evaluation of scores was performed with the 23 full-length sequences (nucleotide and amino acid) of genes described in Table 1. They include genes having at least four different paralogs in one organism, and others with several orthologs in at least four organisms. All of the sequences were compliant with the maximum MSA size that prevents overflow of the M-Coffee size limits. As example of closely-related paralogous genes, the five cytosolic glutamine synthetase isoforms of *Arabidopsis thaliana *(AtGS1) and the four cytosolic glutamine synthetase isoforms of *Oryza sativa *(OsGS1) were included. As an example of orthologous genes, the five genes of mammalian malate dehydrogenase 1 (MDHm), five plant genes of the mitochondrial NAD-dependent malate dehydrogenase (MDHp), and four plant genes of *S*-adenosylmethionine synthetase (SAM) were included. Sequences were aligned with both MultAlin [27] and M-Coffee [26] using default parameters. Average nucleotide identity was over 62% and the amino acid similarity was over 82%. No clear correlation was found among identity/similarity and orthologs/paralogs in these MSAs, and so further testing would not be biased. The terminal portions of the MSAs were automatically removed by AlignMiner in order to analyse only the portions where all sequences were aligned, and so discard the highly "noisy" ends. Hence, uninformative hyper-variable segments were not included in the analysis. However, it should be noted that these hyper-variable regions in nucleotide MSAs could be considered for designing specific probes for Northern and Southern blots.

**Table 1:** Description of sequences used in this work that served to assess the performance of different aspects of AlignMiner; sequences that have been aligned together have a common average identity and similarity values.

Name	Taxon	Organism	Isoform	AC# (nt)	Average identity	AC# (amino acid)	Average similarity
GS1	Plant	*Arabidopsis thaliana*	AtGS1 isoform 1	AF419608	tity	Q56WN1	ity
GS1	Plant	*Arabidopsis thaliana*	AtGS1 isoform 2	AY091101		Q8LCE1	
GS1	Plant	*Arabidopsis thaliana*	AtGS1 isoform 3	AY088312	70%	Q9LVI8	89%
GS1	Plant	*Arabidopsis thaliana*	AtGS1 isoform 4	AY059932		Q9FMD9	
GS1	Plant	*Arabidopsis thaliana*	AtGS1 isoform 5	AK118005		Q86XW5	

GS1	Plant	*Oryza sativa*	OsGS1 isoform 1	AB037664		Q0DXS9	
GS1	Plant	*Oryza sativa*	OsGS1 isoform 2	AB180688	62%	Q0J9E0	82%
GS1	Plant	*Oryza sativa*	OsGS1 isoform 3	AK243037		Q10DZ8	
GS1	Plant	*Oryza sativa*	OsGS1 isoform 4	AB180689		Q10PS4	

MDH-1	Mammalian	*Mus musculus*	MmMDHm	NM_008618		NP_032644	
MDH-1	Mammalian	*Sus scofra*	ScMDHm	MN_213874		NP_999039	
MDH-1	Mammalian	*Rattus norvegicus*	RnMDHm	AF093773	88%	AAC64180	95%
MDH-1	Mammalian	*Homo sapiens*	HsMDHm	NM_005917		NP_005908	
MDH-1	Mammalian	*Equs caballus*	EcMDHm	XM_001494265		XP_001494315	

MDH-1	Plant	*Arabidopsis thaliana*	AtMDHp	AF339684		AAK00366	
MDH-1	Plant	*Prunus persica*	PpMDHp	AF367442		AAL11502	
MDH-1	Plant	*Vitis vinifera*	VvMDHp	AF195869	71%	AAF69802	87%
MDH-1	Plant	*Oryza sativa*	OsMDHp	AF444195		AAM00435	
MDH-1	Plant	*Lycopersicum esculentum*	LsMDHp	AY725474		AAV29198	

SAM-1	Plant	*Arabidopsis thaliana*	AtSAM	AF325061		AAG40413	
SAM-1	Plant	*Triticum aestivum*	TaSAM	EU399630		ABY85789	
SAM-1	Plant	*Zea mays*	ZmSAM	EU960496	65%	ACG32614	92%
SAM-1	Plant	*Gossypum hirsutum*	GhSAM	EF643509		ABS52575	

GDC-H	Plant	*Pinus pinaster*	Photosynthetic	ongoing		NA	
GDC-H	Plant	*Pinus pinaster*	Non-photosynthetic	ongoing		NA	

**Figure 3:**
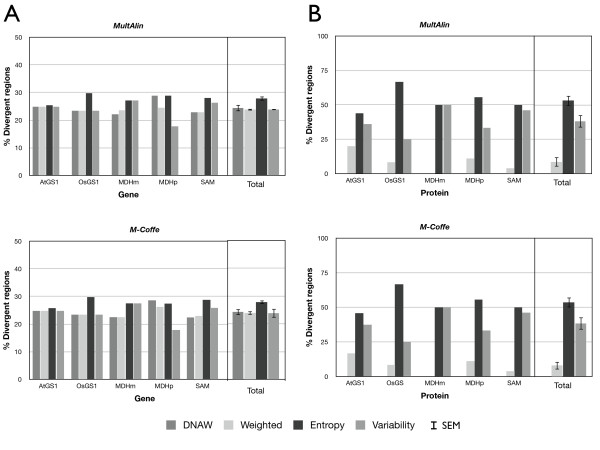
**Distribution of the percentage of divergent regions by alignment and as a total average for nucleotide (A) or amino acid (B) sequences identified with AlignMiner**. Names of the MSAs are explained in Table 1. MultAlin and M-Coffee were used to obtain the input MSAs. SEM, standard error of the mean.

At first, the proportion of divergent regions was compared between MSAs (Figure 3). A percentage was used in order to obtain comparable results, since MSAs of less similar sequences (OsGS1 [paralogs] and SAM [orthologs]) provided more highly-divergent regions than MSAs containing closely-related sequences. In nucleotide MSAs (Figure 3A), Entropy provided the highest number of divergent regions in the five MSAs, while the DNAW, Weighted and Variability methods exhibited variable behaviour. Averaging all the results for a single value with its SEM (standard error of the mean) confirmed the previous result, i.e. the number of divergent regions using Entropy was clearly higher than when using the other methods, among which the percentage was lower and statistically-similar. For amino acid MSAs (Figure 3B), the percentages were more variable among the scoring methods, but Entropy again provided the highest value, while Weighted gave the lowest value in all instances (clearly, it was the most restrictive in both nucleotide and amino acid MSAs). On the other hand, Figure 3B also shows that, when the sequences aligned are very similar (AtGS1, SAM, and MDMm), Entropy and Variability behave similarly with regard to the divergent region percentage, whilst Variability clearly provides a lower number of divergent regions than Entropy. Therefore, Entropy was the method that identified the greatest number of divergent regions for any kind of MSA, while Weighted was revealed to be the most restrictive.

**Figure 4:**
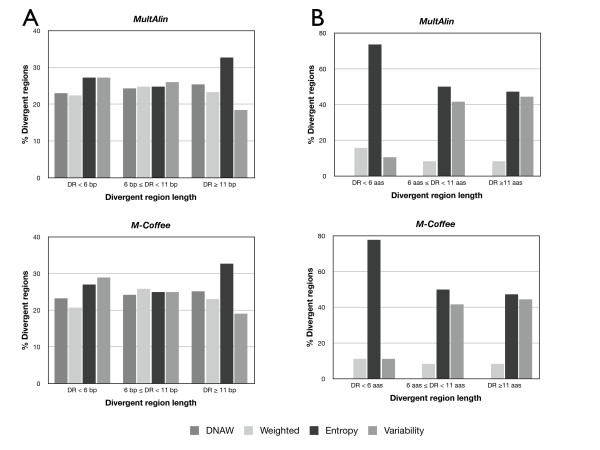
**Distribution of the divergent region percentages by length for DNA (A) or protein (B) MSAs identified with AlignMiner**. Names of the MSAs are explained in Table 1. MultAlin and M-Coffee were used to obtain the input MSAs. DR, divergent region; bp, base pairs; aas, amino acids.

Scoring methods should also be characterised by the region length they determine. Divergent regions were classified by their length in three intervals: less than six positions, between six and 11 positions, or more than 11 positions. In nucleotide MSAs (Figure 4A), it became apparent that Entropy also rendered the longest divergent regions, while all the methods were roughly equivalent for regions below 11 nucleotides. In protein MSAs (Figure 4B), Variability and Entropy behave similarly with respect to identification of divergent regions longer than either six or 11 amino acids, although Entropy in both cases identified a slightly larger number of divergent regions than Variability. Weighted again provided a low number of long divergent regions. However, Entropy provided by far the highest number of divergent regions below six amino acids in length. In conclusion, Entropy seemed to provide not only the highest number of divergent regions, but also the longest ones; in contrast, Weighted was the most restrictive, providing the lowest number of divergent regions, which were also slightly shorter. It could be hypothesised that these differences are due to the fact that Entropy considers only the frequency of symbols (and not the features of the represented object) while Weighted (and DNAW) take into account the properties of the subject amino acid or nucleotides. This is in agreement with the fact that the entropy concept has proven useful in many fields of computational biology, such as sequence logos corresponding to conserved motifs [36] and the identification of evolutionarily-important residues in proteins [3].

**Figure 5:**
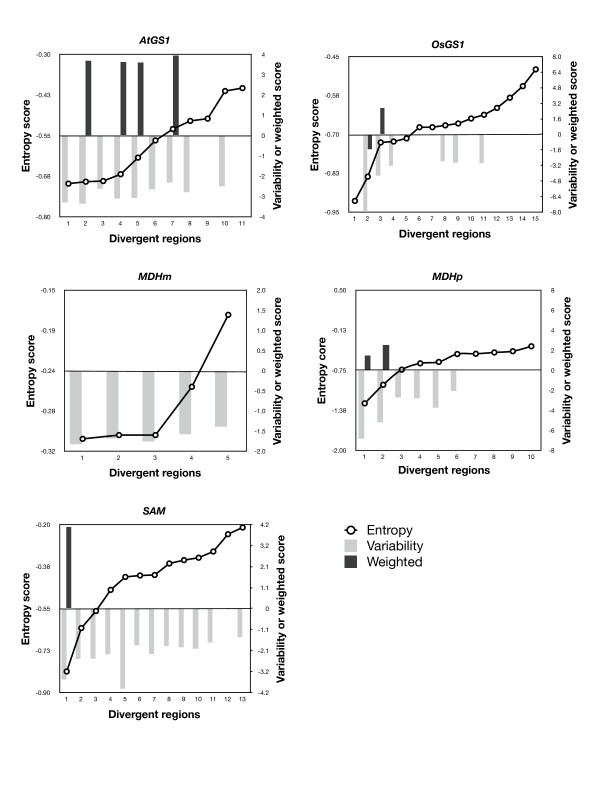
**Distribution of score values of the divergent regions using the three scoring methods **(Entropy, Variability** or **Weighting) **in the five protein MSAs, obtained with M-Coffee**.

Since there seems to be a clear difference in the number and length of divergent regions revealed by the different scoring methods, it could be expected that divergent regions discovered by Variability and Weighted would be included among the regions discovered by Entropy. Figure 5 and Additional file 3 Figure S2 show the divergent regions revealed by Entropy ordered by score for every protein MSA and, superimposed, the scores of the divergent regions revealed by Variability and Weighted. Clearly, the Entropy score included the divergent regions revealed by Variability and Weighted beside other Entropy-specific regions (positions where no column is shown in Figure 5 and Additional file 3 Figure S2). Moreover, the divergent regions revealed by Weighted were often the ones with the highest scores, which is consistent with the fact that this scoring method was the most restrictive. In conclusion, Entropy should be used if a greater number of divergent regions are desired, while Weighted will find use when a small list of only the most significantly-divergent regions is required, and Variability behaves like Entropy when the sequences in the MSA are closely related, but behaves like Weighted in the remainder of cases.

The Entropy scoring method has previously been compared with a scoring method based on phylogenetic theory, such as phastCons [37]. Two different alignments have been used for the comparison. One was a MSA containing the same 1000 nucleotides of four genus *Canis *mitochondrial entries (AC numbers: NC_009686, NC_008092, NC_002008, NC_008093); this alignment only contained 18 divergent positions. The other was the AtGS1 (Table 1) nucleotide MSA. The profile of both scores for both MSAs is shown in Additional file 4 Figure S3. The minimum peaks in the *Canis *MSA analysed with phastCons corresponded to the divergent positions detected in AlignMiner. While phastCons provided different scores for the conserved portions, AlignMiner collapsed them to 0, as described previously. However, in the case of the AtGS1 MSA, where more differences can be found, the situation is the opposite: AlignMiner clearly identified the divergent regions while phastCons collapsed them to 0; moreover, the scores of the divergent regions in this MSA are more highly-negative than in the *Canis *MSA, reflecting the fact that there are more variations in the AtGS1 MSA than in the *Canis *MSA. Therefore, phastCons and AlignMiner appear to be complementary, since phastCons is devoted to conserved fragments while AlignMiner is specialised for divergent regions of MSAs with various levels of similarity. Only when the MSAs share over 99% identity do both algorithms identify the same divergent nucleotides without hesitation.

Figures 3 and 4, as well as Figure 5 and the Additional file 3 Figure S2, show that the AlignMiner results seem to be independent of the alignment algorithm used, since the histograms of M-Coffee are almost identical to those of MultAlin in spite of their different rationales. This is not surprising, because divergent regions are still found among conserved sequences. Therefore, divergent regions found by AlignMiner should not be strongly biased by the alignment algorithm, and this enables users to seed AlignMiner with a MSA generated using their preferred algorithm. This finding is in agreement with other algorithms exploiting the information deposited in each column of a MSA [3]. In accordance with this robustness, only MSAs obtained with M-Coffee will be used from now on.

### In silico proof-of-concept cases

**Figure 6:**
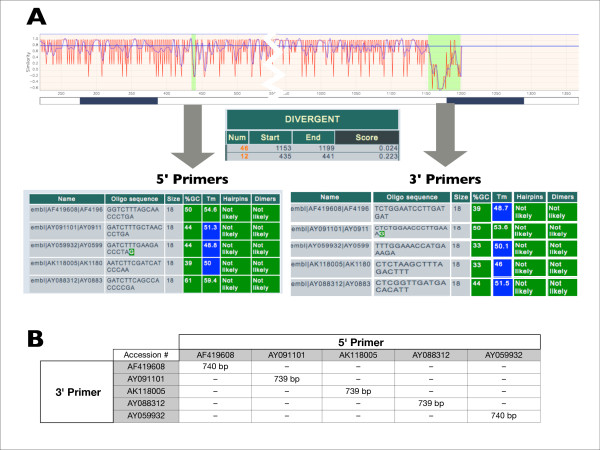
**Use of AlignMiner for designing several specific primer pairs for PCR amplification of the different isoforms of the AtGS1 nucleotide sequence **(A) The 5' and 3' divergent regions obtained with Entropy that were selected for primer design including the characteristic parameters of each region. (B) Results of the *in silico *"PCR amplification" with BioPHP [34] using the different primer pairs. Note that the actual 3' primers are complementary to the sequences shown on the right.

AlignMiner can be used for selecting specific PCR primers that serve to discriminate among closely-related sequences. As an example, divergent regions were obtained for the five *A. thaliana GS1 *isoforms (AtGS1 in Table 1). Since all the scoring methods produce similar results for these sequences (Figure 3), the MSA was inspected with DNAW. The resulting divergent regions were sorted by decreasing score and the best regions (scores 0.223 and 0.024) were selected for primer design (Figure 6A and Table 2) with the help of the primer tool. These primers were shown to selectively amplify each isoform of *GS1 in silico *(Figure 6B), as revealed by "PCR amplification" of the BioPHP suite [35].

**Table 2:** Details of primers designed with AlignMiner to identify specifically by PCR the five *A. thaliana* GS1 genes as well as the two primer pairs that identify the photosynthetic and non-photosynthetic isoforms of *P. pinaster*; note that the 3' (reverse) primer is complementary to the sequence appearing in Figures 6 and 8.

Isoform	Primer	Length	%GC	Tm (°C)	Amplicon size (bp)
GS1.1	5'-GGTCTTTAGCAACCCTGA-3'	18	50	54.6	740
	5'-ATCATCAAGGATTCCAGA-3'	18	39	48.7	

GS1.2	5'-GATCTTTGCTAACCCTGA-3'	18	44	51.3	739
	5'-CTTTCAAGGGTTCCAGAG-3'	18	50	53.6	

GS1.3	5'-AATCTTCGATCATCCCAA-3'	18	39	50	739
	5'-AAAGTCTAAAGCTTAGAG-3'	18	33	46	

GS1.4	5'-GATCTTCAGCCACCCCGA-3'	18	61	59.4	739
	5'-AATGTGTCATCAACCGAG-3'	18	44	51.5	

GS1.5	5'-GATCTTTGAAGACCCTAG-3'	18	44	48.8	740
	5'-TCTTTCATGGTTTCCAAA-3'	18	33	50.1	

Photosyntetic isoform	5'-AGTGCGCATTAAGGACCCATCA-3'	22	50	61	177
	5'-ACACACTGGCTTCCACAATAGG-3'	22	50	59.4	

Non-photosynthetic isoform	5'-ACAGATGATCTAGGACATGC-3'	20	45	52	169
	5'-CACTTATTTGCACTTGAAGG-3'	20	40	52.6	

**Figure 7:**
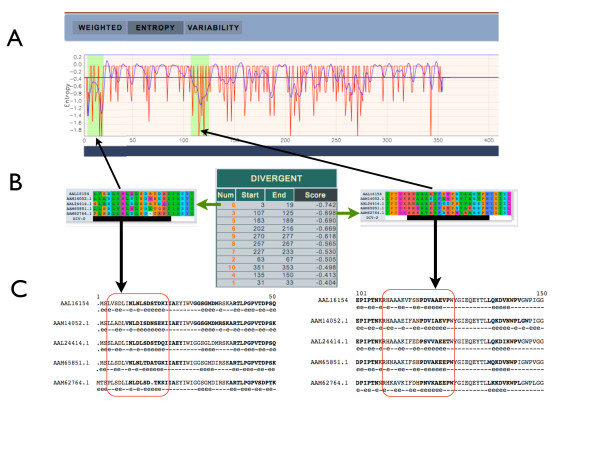
**Correlation between the most divergent amino acid sequences and antigenicity of the AtGS1 protein MSA**. (A) Similarity plot obtained using the Entropy method; the most divergent regions being are highlighted. (B) Aligned sequences for the two divergent regions together (underlined in black) and their score in relation to other divergent regions. (C) Localisation of each divergent region in the alignment where: (i) nucleotides in bold are the predicted epitopes for B-cells; (ii) an "e" denotes predicted solvent accessibility for this position; and (iii) red-boxed amino acids correspond to the sequence of the matching divergent region. It is clearly seen that divergent sequences overlap with the predicted epitopes and the solvent-accessible amino acids.

Identification of divergent regions among proteins can also be performed. It may be hypothesised that the most divergent regions could be epitopes for production of specific, even monoclonal, antibodies that can serve to distinguish very closely-related protein isoforms. As an example, the five glutamine synthetase (GS1) enzyme isoforms of *A. thaliana *(AtGS1, Table 1) were aligned with MultAlin using default parameters. The Entropy scoring method was used since it identified the longest divergent regions (Figure 4). The resulting divergent regions were sorted by score and the best ones were selected (Figure 7B). Each GS1 sequence was additionally inspected for solvent-accessible positions and highly antigenic regions using the SCRATCH Protein Predictor Web suite [38]. It appeared that the most highly-divergent Entropy-derived regions corresponded to the most solvent-accessible and most antigenic portions of the protein (Figure 7C). These sequences can then be used to challenge mice or rabbits and obtain specific antibodies against any one of the aligned sequences.

### Experimental case study of divergent regions in a nucleotide MSA

**Figure 8:**
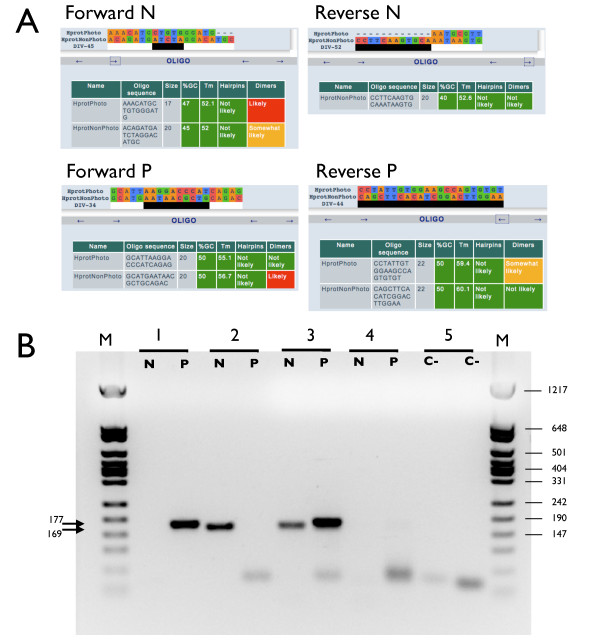
**Analysis of two *Pinus pinaster *gene isoforms. The specific primer pair for the photosynthetic isoform is identified by a "P" and for the non-photosynthetic isoform by an "N" **(A) Predicted sequence and properties of the two primer pairs designed for specific identification of each isoform. (B) PCR analysis using the previously-predicted primers. Table 2 includes the expected amplicon size using these primer pairs. The template in the different lanes is: cDNA for the photosynthetic isoform (lanes 1), cDNA for the non-photosynthetic isoform (lanes 2), cDNA synthesised from total mRNA extracted from *Pinus pinaster *(lanes 3), *Pinus pinaster *genomic DNA (lanes 4), and negative controls (lines 5), which do not contain any DNA. Lanes M are molecular weight markers (vector pFL61 digested with *Hpa *II). Arrows indicate the specific amplification bands. DNA sizes are given in base pairs.

AlignMiner was tested for its efficacy in the design of PCR primers in a real laboratory setting. Two isoforms of a *Pinus pinaster *gene, one from photosynthetic tissue and one from non-photosynthetic tissue (Table 1) were analysed. Sequences were aligned with MultAlin using default settings. The resulting alignment was loaded into AlignMiner and divergent regions were identified with the Weighted score in obtain a small list of the most divergent regions. This enabled the design of specific primers for the photosynthetic and non-photosynthetic isoforms (Figure 8A, Table 2). PCR amplification with these primers using different DNAs as template (Figure 8B) confirmed that each primer pair amplified only the isoform for which it was designed, without any cross-amplification, using as template either total cDNA (Figure 8B, lanes 3) or specific cDNA inserted into a plasmid (Figure 8B, lanes 1 and 2). Since primers pairs are expected to span exon-exon junctions, no amplification was observed using genomic DNA (Figure 8B, lanes 4). These results suggested that the algorithm had correctly identified a divergent region, and that the primers were correctly designed and worked as predicted by the software.

## Conclusions

AlignMiner serves to fill the gap in bioinformatic function for the study of sequence divergence in MSAs containing closely-related sequences. In contrast to other software [15,18,39], it is not intended for the design of primers for high-throughput analysis but for the of study particular cases where very closely-related sequences must be distinguished in order to avoid cross-reaction. AlignMiner is able to identify conserved/divergent regions with respect to a consensus sequence or to a "master" sequence. It can even be used to identify putative DNA probes for blotting hybridisation that correspond to the hyper-variable regions at each MSA end. Our tests demonstrate that the predictions of AlignMiner are not markedly affected by the mode of MSA generation. This is mainly attributable to the fact that the MSAs comprised highly similar sequences, and most differences among MSA algorithms involve divergent regions [40]. In this study, the degree of similarity among sequences did not appear to qualitatively affect the results. While Entropy provided the highest number of divergent regions of the longest size, Weighted provided only a small set of the most divergent regions. Additionally, AlignMiner was found to be complementary to the phastCons algorithm [37], since the former reinforces the differences and the latter, the similarities. Wet and dry laboratory experiments showed that AlignMiner can be used to provide specific primers for PCR amplification of one gene among a gene family of orthologs and paralogs, as well as to select protein epitopes for antibody production. Moreover, use of this software confirmed that divergent regions in protein alignments can be viewed as putative specific antigenic sequences.

The calculations in AlignMiner have been optimised in order to reduce execution times. In contrast to other more static Web-based applications [15-17,19,39], the AlignMiner interface is highly interactive, using emergent Web technologies without third party solutions in order to resemble a stand-alone application. This renders the interactivity highly dependent on the computer capabilities and browser implementation. Its implementation as a Web tool enables users to inspect their results on different computers, even those with different operating systems. The data flexibility means that it can handle most MSA formats, with each MSA obtained from orthologous and/or paralogous sequences, and can be saved in standard formats (PNG for images and GFF for data). We hope that AlignMiner will save researchers time when designing PCR primers, probes, and linear epitopes. We are also open to suggestions from the scientific community towards further development of AlignMiner. Institutions wishing to host mirrors of AlignMiner are encouraged to contact the authors.

## Availability and requirements

**Project name **AlignMiner. No license or account is needed.

**Operating systems **Platform-independent

**Programming languages **Perl for the algorithm; Ajax and HTML for the Web interface.

**Other requirements **A Web browser supporting JavaScript and Ajax (preferably Mozilla Firefox or Safari) is required to use the public Web server. For installing AlignMiner, BioPerl, JSON, Log::Log4perl and Math:FFT Perl modules are required.

## List of abbreviations

MSAs: multiple sequence alignments; PCR: polymerase chain reaction; dNTP: deoxynucleotide triphosphate; SEM: standard error of the mean; FFT: fast Fourier transform; DR: divergent region; bp: base pairs; aas: amino acids; AtGS1: cytosolic glutamine synthetase isoforms of *Arabidopsis thaliana*; OsGS1: cytosolic glutamine synthetase of *Oryza sativa; *MDHm: mammalian malate dehydrogenase 1; MDHp: plant mitochondrial NAD-dependent malate dehydrogenase; SAM: plant *S*-adenosylmethionine synthetase.

## Competing interests

The authors declare that they have no competing interests.

## Authors' contributions

DG designed the interface and implemented the software. RB conducted the *in silico *testing. DPV conducted the experimental testing. FRC supervised the *in silica *and experimental testing, and helped in the manuscript writing. MGC conceived the study and wrote the manuscript. All authors read and approved the final manuscript.

## Supplementary Material

Additional file 1 **Figure S1**. Examples of primer design using the primer tool of AlignMiner. In a good-quality set of oligonucleotides (left), all properties have a green background. When several primer properties are not in agreement with the characteristics stated in the text (right), they are marked in red (very bad), orange (adequate only) or blue (melting point is too low). In such a case, the sequence window must be moved around the divergent region (extending or narrowing it) in order to find a "green" primer; otherwise other divergent/conserved regions should be considered. Click here for file

Additional file 2 **Table S1**. Details of the REST elements available, and the relevant instructions for invoking AlignMiner as a Web service. Click here for file

Additional file 3 **Figure S2**. Distribution of score values of the divergent regions using the three scoring methods (Entropy, Variability or Weighting) in the five protein MSAs obtained with MultAlin. Click here for file

Additional file 4 **Figure S3**. Scoring comparison provided by phastCons and AlignMiner with the MSAs of AtGS1 (left) and a highly-conserved fragment of 1000 nucleotides (right) from four different *Canis *mitochondrial DNAs. Click here for file
