# Supplementary material for: AlignMiner: a Web-based tool for detection of divergent regions in multiple sequence alignments of conserved sequences
Source: Algorithms Mol Biol. 2010 Jun 2;5:24. doi: 10.1186/1748-7188-5-24 (PMC2902484; doi:10.1186/1748-7188-5-24)
Supplement: Additional file 1 — Figure S1. Examples of primer design using the primer tool of AlignMiner. In a good-quality set of oligonucleotides (left), all properties have a green background. When several primer properties are not in agreement with the characteristics stated in the text (right), they are marked in red (very bad), orange (adequate only) or blue (melting point is too low). In such a case, the sequence window must be moved around the divergent region (extending or narrowing it) in order to find a "green" primer; otherwise other divergent/conserved regions should be considered. [file 1748-7188-5-24-S1.PDF]

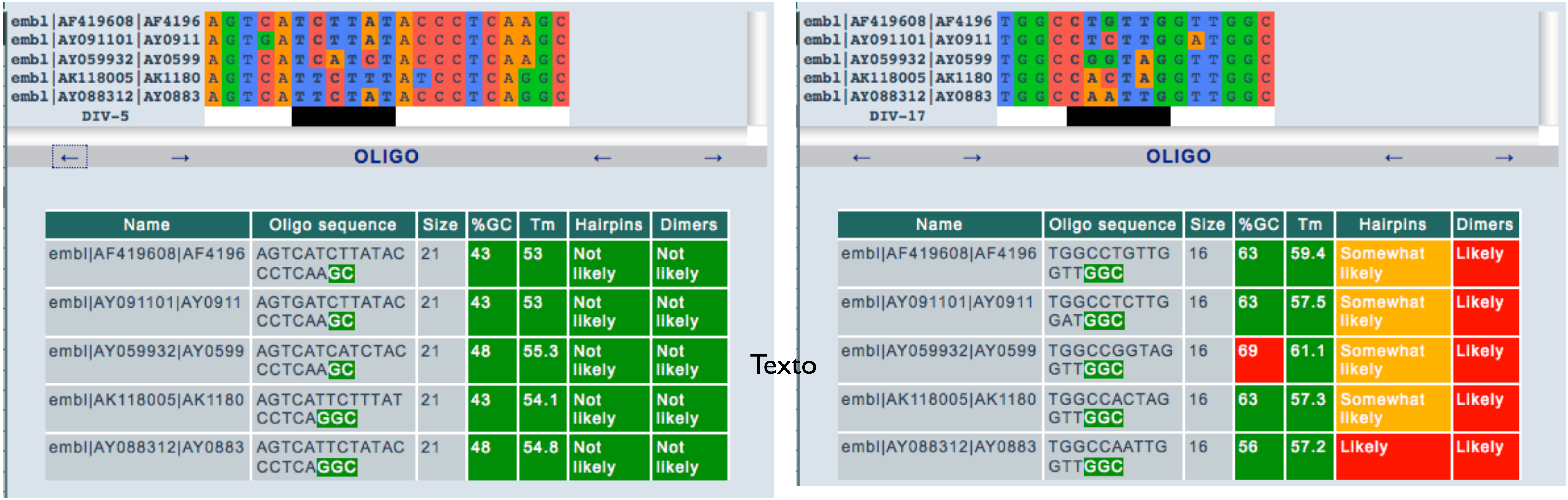

Good

Bad

Figure S1: Examples of primer design using the primer tool of AlignMiner. In a good-quality set of oligonucleotides (left), all properties have a green background. When several primer properties are not in agreement with the characteristics stated in the text (right), they are marked in red (very bad), orange (adequate only) or blue (melting point is too low). In such a case, the sequence window must be moved around the divergent region (extending or narrowing it) in order to find a ``green'' primer; otherwise other divergent/conserved regions should be considered.
