# Supplementary material for: AlignMiner: a Web-based tool for detection of divergent regions in multiple sequence alignments of conserved sequences
Source: Algorithms Mol Biol. 2010 Jun 2;5:24. doi: 10.1186/1748-7188-5-24 (PMC2902484; doi:10.1186/1748-7188-5-24)
Supplement: Additional file 2 — Table S1. Details of the REST elements available, and the relevant instructions for invoking AlignMiner as a Web service. [file 1748-7188-5-24-S2.PDF]

Table S1: Details of the REST elements available, and the relevant instructions for invoking AlignMiner as a Web service

<table>
<tr><th>HTTP VERB</th><th>URL</th><th>FIELDS</th><th>Description</th></tr>
<tr><td>POST</td><td>http://www.scbi.uma.es/ingebiol//commands/am/jobs/0/stage/1.json</td><td>api_login_key=your@email.com
alignment_file_field= @<file_path>
job_name_field=<any text>
kalign_field=<0 | 1>
master_field=<NONE | seqname>
align_start_field=<a number>
align_end_field=<a number></td><td>Sending a new job and returns either errors or the JOB_ID if it was successfully uploaded</td></tr>
<tr><td>GET</td><td>http://www.scbi.uma.es/ingebiol/commands/am/jobs.json</td><td>api_login_key=your@email.com</td><td>Retrieving the complete job list</td></tr>
<tr><td>GET</td><td>http://www.scbi.uma.es/ingebiol/commands/am/jobs/<JOB_ID></td><td>api_login_key=your@email.com</td><td>Obtaining the status of a job</td></tr>
<tr><td>GET</td><td>http://www.scbi.uma.es/ingebiol/downloads/am/<JOB_ID></td><td>api_login_key=your@email.com</td><td>Downloading the complete result set of a job</td></tr>
<tr><td>GET</td><td>http://www.scbi.uma.es/ingebiol/downloads/am/<JOB_ID>/<ONE_FILE_OR_FOLDER></td><td>api_login_key=your@email.com</td><td>Downloading only one file or folder from the set of job results</td></tr>
<tr><td>DELETE</td><td>http://www.scbi.uma.es/ingebiol/downloads/am/<JOB_ID>.json</td><td>api_login_key=your@email.com</td><td>Deleting the job JOB_ID</td></tr>
</table>
