# Supplementary material for: AlignMiner: a Web-based tool for detection of divergent regions in multiple sequence alignments of conserved sequences
Source: Algorithms Mol Biol. 2010 Jun 2;5:24. doi: 10.1186/1748-7188-5-24 (PMC2902484; doi:10.1186/1748-7188-5-24)
Supplement: Additional file 3 — Figure S2. Distribution of score values of the divergent regions using the three scoring methods (Entropy, Variability or Weighting) in the five protein MSAs obtained with MultAlin. [file 1748-7188-5-24-S3.PDF]

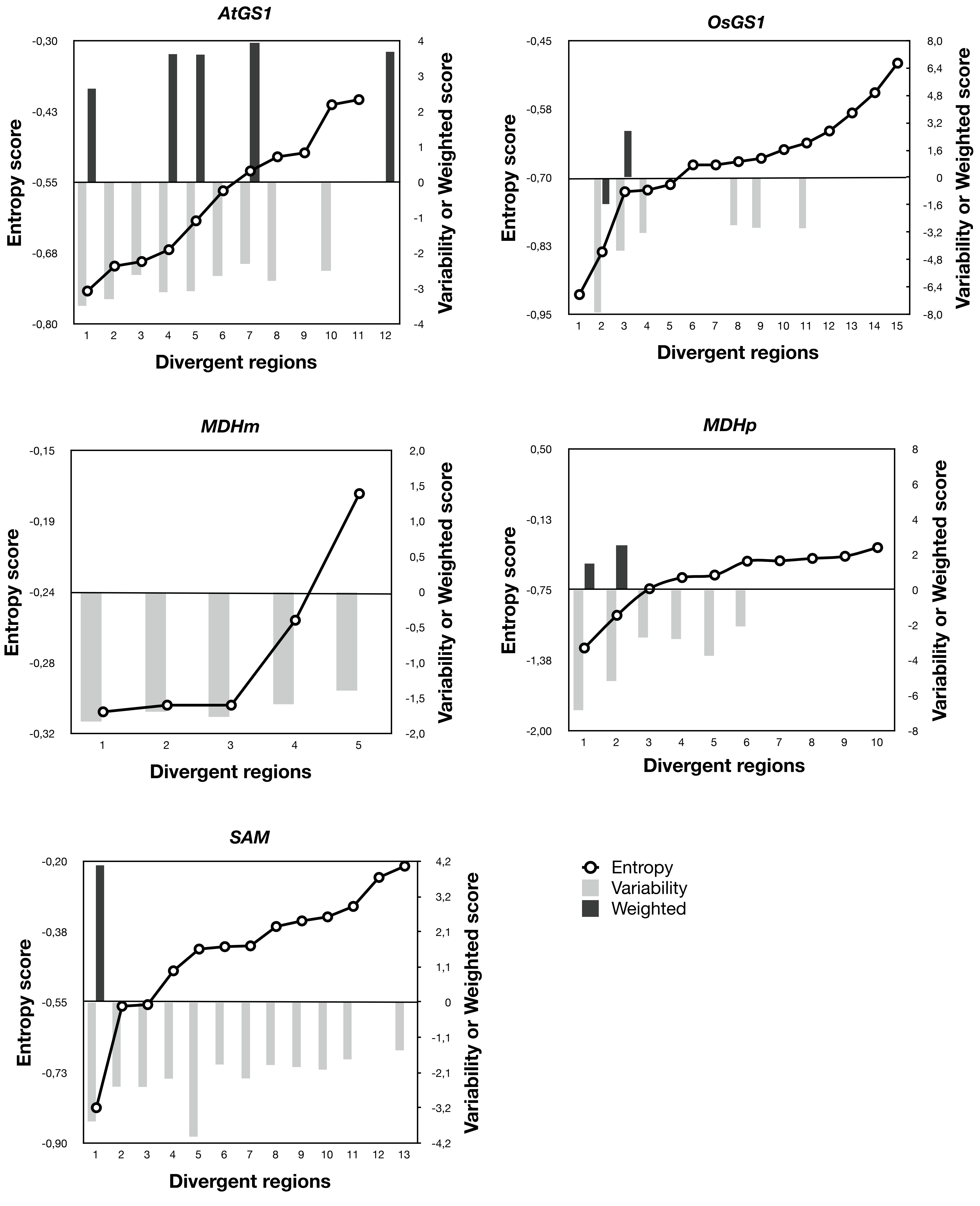

Figure S2: Distribution of score values of the divergent regions using the three scoring methods (`Entropy`, `Variability` or `Weighting`) in the five protein MSAs obtained with MultAlin.
