# Supplementary material for: AlignMiner: a Web-based tool for detection of divergent regions in multiple sequence alignments of conserved sequences
Source: Algorithms Mol Biol. 2010 Jun 2;5:24. doi: 10.1186/1748-7188-5-24 (PMC2902484; doi:10.1186/1748-7188-5-24)
Supplement: Additional file 4 — Figure S3. Scoring comparison provided by phastCons and AlignMiner with the MSAs of AtGS1 (left) and a highly-conserved fragment of 1000 nucleotides (right) from four different Canis mitochondrial DNAs. [file 1748-7188-5-24-S4.PDF]

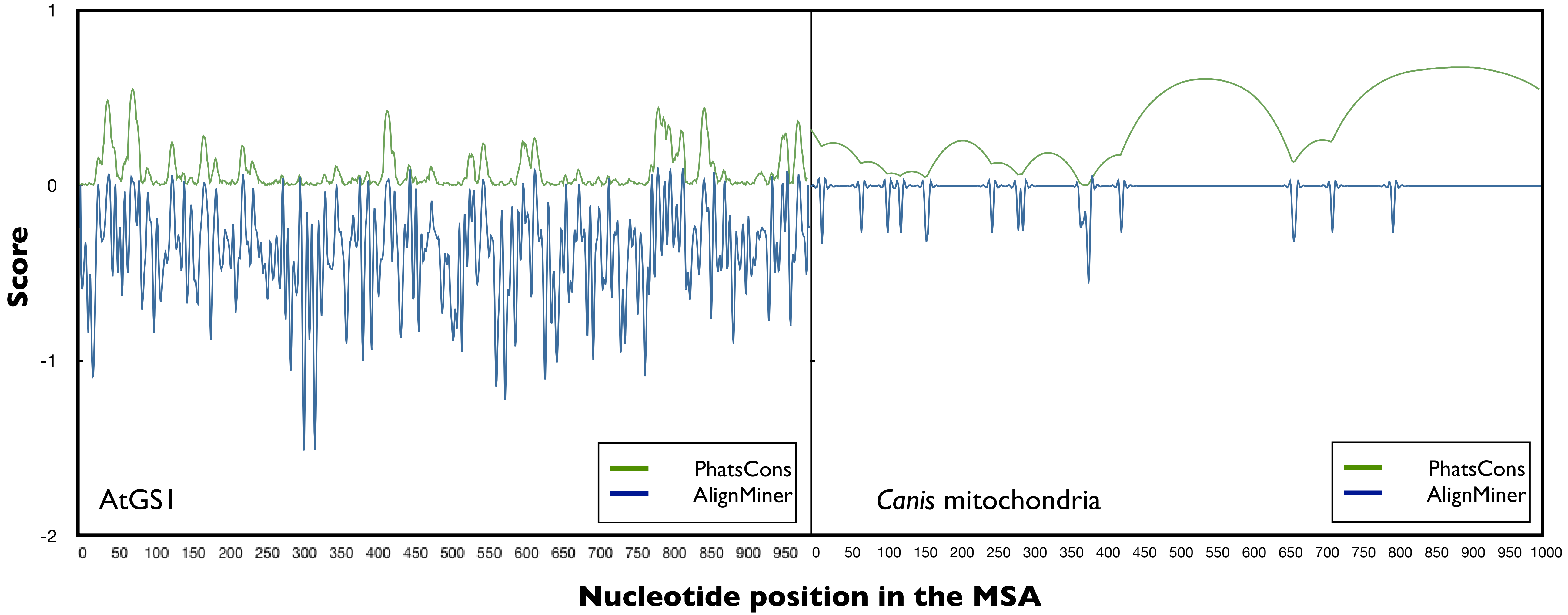

Figure S3: Scoring comparison provided by phastCons and AlignMiner with the MSAs of AtGS1 (left) and a highly-conserved fragment of 1000 nucleotides (right) from four different *Canis* mitochondrial DNAs.
